# It takes two: Seasonal variation in sexually dimorphic weaponry results from divergent changes in males and females

**DOI:** 10.1002/ece3.5136

**Published:** 2019-04-16

**Authors:** Whitney L. Heuring, Melissa Hughes

**Affiliations:** ^1^ Department of Biology & Grice Marine Laboratory College of Charleston Charleston South Carolina; ^2^ Present address: Conservation and Science Department Arizona Center for Nature Conservation/Phoenix Zoo Phoenix Arizona

**Keywords:** female aggression, intrasexual selection, sexual dimorphism, social selection, weapons

## Abstract

Sexually dimorphic weaponry often results from intrasexual selection, and weapon size can vary seasonally when costs of bearing the weapon exceed the benefits outside of the reproductive season. Weapons can also be favored in competition over nonreproductive resources such as food or shelter, and if such nonreproductive competition occurs year‐round, weapons may be less likely to vary seasonally. In snapping shrimp (*Alpheus angulosus*), both sexes have an enlarged snapping claw (a potentially deadly weapon), and males of many species have larger claws than females, although females are more aggressive. This contrasting sexual dimorphism (larger weaponry in males, higher aggression in females) raises the question of whether weaponry and aggression are favored by the same mechanisms in males and females. We used field data to determine whether either sex shows seasonal variation in claw size such as described above. We found sexual dimorphism increased during the reproductive season due to opposing changes in both male and female claw size. Males had larger claws during the reproductive season than during the nonreproductive season, a pattern consistent with sexual selection. Females, however, had larger claws during the nonreproductive season than during the reproductive season—a previously unknown pattern of variation in weapon size. The observed changes in female weapon size suggest a trade‐off between claw growth and reproduction in the reproductive season, with investment in claw growth primarily in the nonreproductive season. Sexually dimorphic weaponry in snapping shrimp, then, varies seasonally due to sex differences in seasonal patterns of investment in claw growth, suggesting claws may be advantageous for both sexes but in different contexts. Thus, understanding sexual dimorphisms through the lens of one sex yields an incomplete understanding of the factors favoring their evolution.

## 1 INTRODUCTION

Sexual selection often leads to sexual dimorphisms in behavior, ornamentation, or weaponry (Darwin, 1871). Nonetheless, the presence of sexually dimorphic traits is not by itself direct evidence of sexual selection, as other processes can also result in sexual dimorphisms (Shine, 1989). For example, sexual dimorphisms in weaponry are typically attributed to sexual selection—in particular, intrasexual selection, in which individuals of one sex (usually males) compete with other members of the same sex for access to mates or resources advantageous for reproduction (Andersson, 1994; Emlen, 2008). However, aggressive behaviors and weaponry can be advantageous in contexts other than same‐sex competition for reproductive resources, such as defense of shelter in the nonreproductive season or competition over food (Lyon & Montgomerie, 2012; Tobias, Montgomerie, & Lyon, 2012), and if the benefits of such competition differ between the sexes, such nonreproductive competition could favor sexual dimorphisms in weaponry or aggression as well. Here, we explored seasonal variation in the size of a sexually dimorphic weapon to determine whether seasonal changes in either sex were consistent with advantages associated with reproductive or nonreproductive competition.

Seasonal variation in weapon size is a relatively underutilized variable that has the potential to provide insight into functional advantages of weaponry, especially in species for which direct evidence of impacts on reproductive success is difficult to obtain. Weaponry under sexual selection would be predicted to be larger during reproductive season, as the benefits of such weapons relative to the costs of bearing them would be higher during the reproductive season. For example, crayfish show sexual dimorphism in claw size only during the reproductive season; for males, the molt following the reproductive season results in increases in body (carapace) length that are much greater than increases in claw size, resulting in a reduction in claw/body size ratios (Stein, 1976). Greater dimorphism during the reproductive season in traits used in male–male competition has been documented in hermit crabs (claw size; Yasuda, Otoda, Nakano, Takiya, & Koga, 2017) and Tupinambis lizards (jaw muscles; Naretto, Gardozo, Blengini, & Chiaraviglio, 2014). In contrast, weaponry used in competition for nonreproductive resources would be predicted to show no seasonal variation (as seen in female crayfish, Stein, 1976), as the benefits of nonreproductive competition are likely to accrue year‐round. Thus, predicted patterns for seasonal variation in weapon size differ for weapons used primarily for reproductive versus nonreproductive competition.

Alpheid snapping shrimp (*Alpheus* spp.) are small, benthic, decapod crustaceans bearing an extraordinarily large snapping claw (claw length in both sexes is typically 35%–55% of body length; e.g., see Knowlton & Keller, 1982, this study), which is used as a weapon in aggressive interactions (Knowlton & Keller, 1982; Nolan & Salmon, 1970) and as a visual signal (Hazlett & Winn, 1962; Hughes, 1996; Nolan & Salmon, 1970; Schein, 1975,1977). Snapping shrimp continue to grow throughout their adult lives with larger individuals bearing larger claws, and claw size is sexually dimorphic in some taxa, with males having larger claws relative to body size (i.e., steeper slopes in regressions of claw size on body size) than females (Hughes, 1996; Hughes, Williamson, Hollowell, & Vickery, 2014; Knowlton, 1980; Schein, 1975). Although male shrimp have a larger weapon than similar‐sized females, females are more aggressive (produce more snaps) in same‐sex interactions, and are more likely to have contests involving lethal aggression (Hughes et al., 2014; Knowlton & Keller, 1982).

In many animals, weaponry and aggression in males are typically attributed to intrasexual selection, while in females these traits are sometimes attributed to selection in nonreproductive contexts: Females, for example, may benefit from winning aggressive contests over ecological rather than reproductive resources (Tobias et al., 2012). Nonetheless, there is growing recognition that female–female competition can be favored in a reproductive context as well (Rosvall, 2008,2011). In snapping shrimp, however, direct evidence for the effect of weapon size on reproductive success in either sex is lacking, and the indirect evidence that is available does not support a reproductive advantage for larger claws in males. For example, in *Alpheus heterochaelis*, females (rather than males) compete for larger mates (Rahman, Dunham, & Govind, 2004). In *Alpheus angulosus*, males with larger claws are not more likely to be paired or have a pair mate brooding eggs than males with smaller claws (Hughes et al., 2014), although males do appear to engage in selective mate guarding of females (Mathews, 2002), and larger weapons may be advantageous in this context. Thus, in snapping shrimp, although males have larger claws than females, it is not clear whether males benefit from larger claws in reproductive contexts.

Snapping shrimp molt approximately once per month; each molt presents an opportunity for differential investment in growth of the claw versus growth in overall body size or other structures (Stein, 1976). Seasonal variation in claw size, then, may provide insight into the relative roles of competition for reproductive versus competition for nonreproductive resources in favoring larger weapon size within each sex. If larger weaponry is primarily advantageous in contests for reproductive resources, claws would be expected to be relatively larger during the reproductive season than the nonreproductive season; in contrast, if larger weaponry is primarily advantageous in contests over nonreproductive resources, the relationship between claw and body size would not be expected to show seasonal variation.

## 2 MATERIALS AND METHODS

### 2.1 Collection and measurement

Snapping shrimp (*A. angulosus*) were collected from May 2014 to February 2016 in mudflat intertidal habitats with oyster shell at three sites in Charleston Harbor, Charleston, SC, USA: Grice Marine Laboratory (32.7524°N, 79.8975°W), Pickett Bridge Recreation Area (32.7695°N, 79.8620°W), and Melton Peter Demetre Park (32.7538°N, 79.9154°W). Shrimp were collected at low tide by flipping over rocks or oyster rubble covering small tide pools (generally <30 cm in diameter), digging into the mud, and using a net to capture the shrimp. Typically, no more than two shrimp (one male and one female) are found within each tide pool; shrimp collected from the same tide pool were considered paired.

Within 2 days of capture, we determined the sex of all shrimp (*n* = 1,555) and measured body (rostrum to telson) and claw (base of propodus to tip of dactyl) lengths to the nearest millimeter using a ruler, as this method minimized handling time and likelihood of claw autotomy. To verify the accuracy of the ruler measurements, claw lengths were measured for a subset of shrimp using calipers (*n* = 361 shrimp measured; mean ± *SE* difference between ruler and caliper measures: 0.005 ± 0.001 cm) and ImageJ (*n* = 614 shrimp photographed; mean ± *SE* difference between ruler and ImageJ measures: 0.006 ± 0.002 cm).

### 2.2 Seasonal variation in sexually dimorphic weaponry

**Figure A1:**
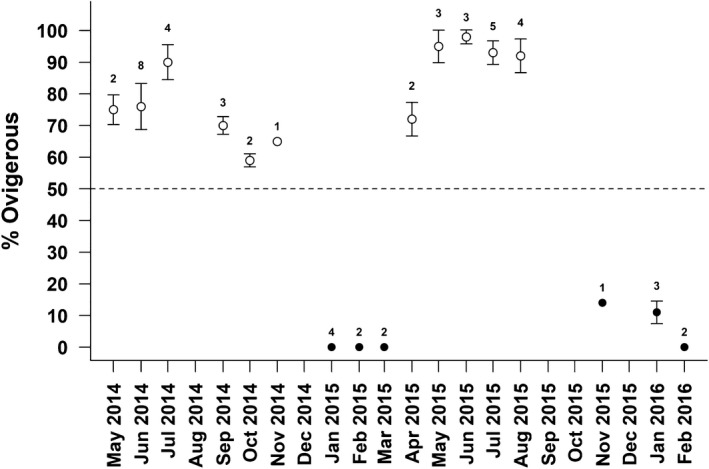
Percent of females carrying eggs (ovigerous) for each month; numbers indicate the number of collection dates within each month. Open symbols are months defined as reproductive season; closed symbols indicate nonreproductive season

**Figure A2:**
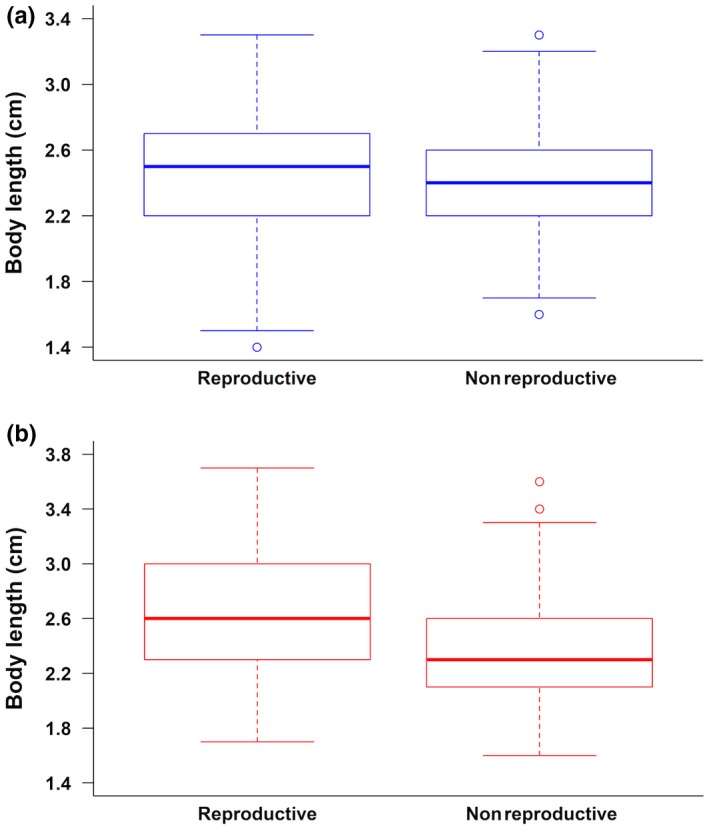
Body sizes of individuals collected during the reproductive and nonreproductive seasons: (a) males; (b) females

The reproductive season was operationally defined as months in which at least 50% of females were reproductively active (carrying eggs); months with 20% or fewer were defined as the nonreproductive season (in no month was the percent reproductively active females between 20% and 50%; see Appendix 1, Figure A1). The body length of males collected during the reproductive and nonreproductive seasons did not differ; females collected during the reproductive season were larger than those collected in the nonreproductive season, primarily due to changes in the number of very large individuals (Appendix 1, Figure A2).

To determine whether either male or female claw size varies seasonally, we performed two analyses; from both, shrimp with missing or regenerating claws were excluded. First, we calculated the ratio of claw length/body length for each shrimp and compared claw length/body length ratios between reproductive (males *n* = 543, females *n* = 541) and nonreproductive (males *n* = 237, females *n* = 234) seasons for each sex using nested ANOVAs (collection date nested in season). Trait/body size ratios can be useful to compare sizes of allometric traits and have been used previously in snapping shrimp (Knowlton & Keller, 1982); however, they can be misleading if the intercepts of regressions of trait on body size deviate from zero (Curran‐Everett, 2013). While in the case of snapping shrimp claws this effect is likely to be small (see Hughes et al., 2014, this study), we also performed ANCOVAs for males (*n* = 780) and females (*n* = 775) of claw size by body size with season as a factor. We report the results of both log‐transformed and untransformed data to facilitate comparisons with previous studies (Hughes et al., 2014; Knowlton, 1980; Schein, 1975).

Previous work (Hughes et al., 2014) found that females carrying eggs at capture had smaller residual claw sizes (residuals from the regression of claw size on body size) than females not carrying eggs at capture, suggesting that larger claws may limit the frequency of female reproduction. However, this analysis included females collected throughout the year and thus would be confounded by any seasonal variation in claw size. We repeated this analysis here, comparing residual claw sizes of females with and without eggs at capture, using only females collected in the reproductive season.

All statistical analyses were performed using R 3.1.2 (R Core Team, 2014). We examined residuals for normality and confirmed homoscedasticity for parametric analyses; in cases where data did not meet assumptions of parametric analyses, nonparametric analyses were used.

## 3 RESULTS

**Figure 1:**
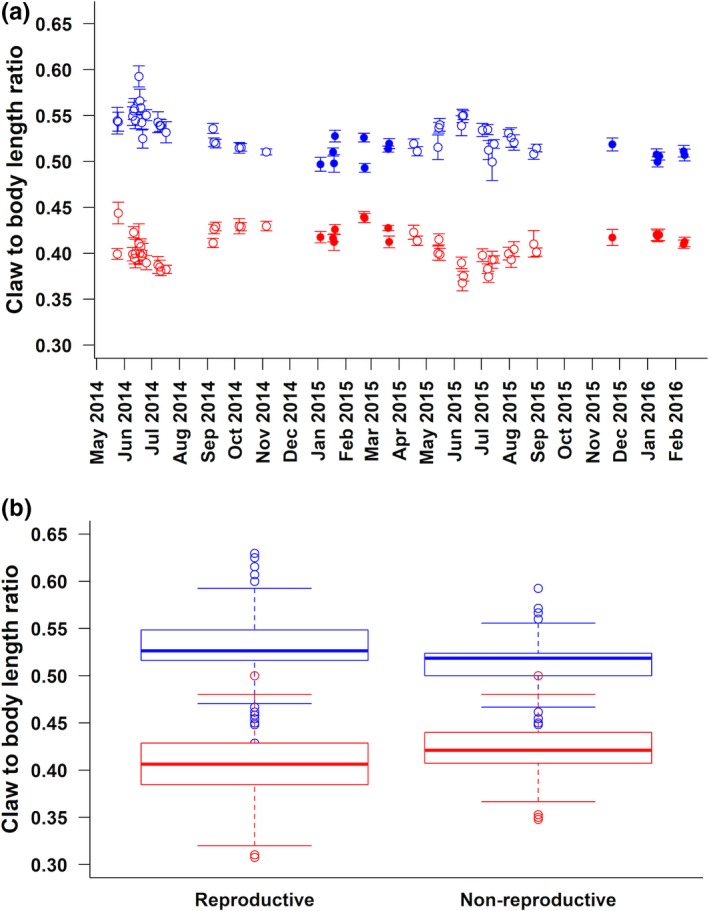
Seasonal variation in claw/body ratio: (a) Mean ± *SE* claw/body ratio across collections within months for males (blue, above) and females (red, below); open circles are reproductive season (*n* = 40), and closed circles are nonreproductive season (*n* = 14); (b) Claw/body ratio for males (blue, above) and females (red, below) in the reproductive and nonreproductive seasons. Boxes indicate interquartile range; line indicates median; whiskers of box indicate range of values within 1.5 * interquartile range above and below the interquartile range; open circles indicate outliers with values >1.5 * interquartile range above and below interquartile range

We found cyclic seasonal variation in sexual dimorphism in claw/body size across the 22‐month study period, resulting from seasonal variation in both male and female claw/body size (Figure 1a). Claw/body ratio for males was significantly higher in reproductive season than in the nonreproductive season (*F*_(1,54)_ = 20.550, *p* < 0.001), while claw/body ratio for females was significantly higher in nonreproductive season than in the reproductive season (*F*_(1,54)_ = 13.873, *p* < 0.001); these divergent patterns resulted in claw/body ratios that were more similar between the sexes during the nonreproductive season than during the reproductive season (Figure 1b).

**Figure 2:**
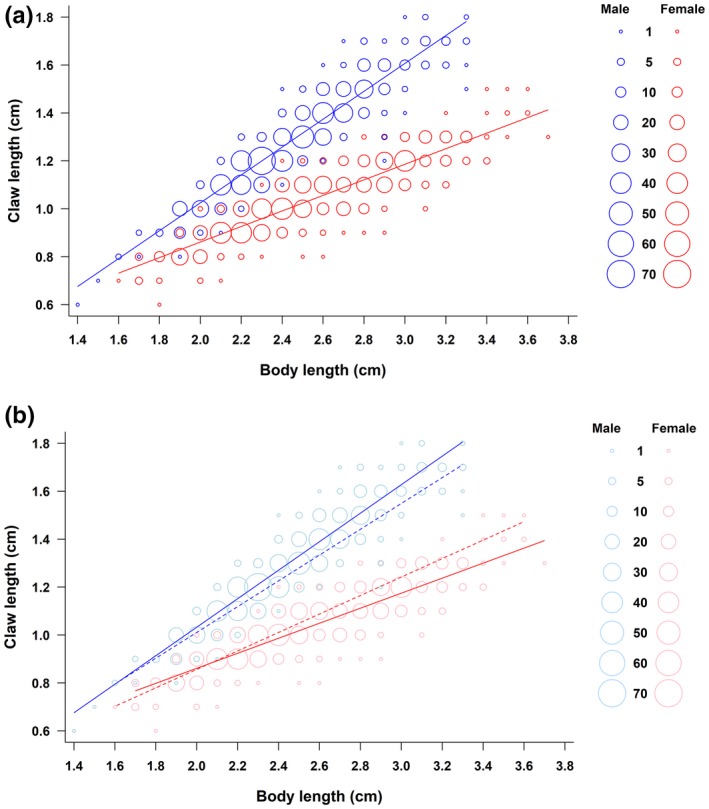
Claw allometry for males (blue, above) and females (red, below): (a) all males (*n* = 780) and females (*n* = 775), symbol size indicates number of observations; (b) regression lines for reproductive (solid lines; male *n* = 543, female *n* = 541) and nonreproductive (dashed lines; male *n* = 237, female *n* = 234) seasons

In both males and females, there were significant relationships between claw length and body length (males: *F*_(1,776)_ = 5991.95, *p* < 0.001; females: *F*_(1,771)_ = 3170.79, *p* < 0.001; Figure 2a). The ANCOVA yielded the same results as the claw/body ratio analysis: We found a significant interaction between season and the slope of the relationship between claw size and body size in both sexes (males: *F*_(1,776)_ = 11.85, *p* < 0.001; females: *F*_(1,771)_ = 27.95, *p* < 0.001; Figure 2b). For males, the slope was steeper during reproductive season than nonreproductive season (Figure 2b); the log‐transformed slopes were >1 in both seasons (Table 1). Females had the opposite pattern, with steeper slopes in nonreproductive season than in reproductive season; log‐transformed slopes for females were <1 in both reproductive and nonreproductive seasons (Table 1). In both seasons, the slope of the relationship between claw and body length was greater in males than females, as indicated by nonoverlapping 95% confidence intervals (Table 1).

**Table 1:** Linear and log‐transformed relationships between claw and body size

Sex	Season (*n*)	Slope (95% CI)	Intercept (95% CI)	*R* ^2^	Log–log slope (95% CI)
Males	Reproductive (543)	0.595 (0.577, 0.613)	−0.157 (−0.203, −0.111)	0.882	1.142 (1.108, 1.176)
	Nonreproductive (237)	0.538 (0.514, 0.563)	−0.065 (−0.125, −0.006)	0.890	1.059 (1.012, 1.106)
Females	Reproductive (541)	0.314 (0.299, 0.328)	0.234 (0.195, 0.273)	0.764	0.773 (0.736, 0.809)
	Nonreproductive (234)	0.385 (0.366, 0.405)	0.086 (0.040, 0.131)	0.871	0.932 (0.885, 0.978)

We found no difference in residual claw size between females with and without eggs at capture during the reproductive season (Mann–Whitney *U* test: brooding eggs *n* = 452, not brooding eggs *n* = 89, *W* = 19,266, *p* = 0.5293).

## 4 DISCUSSION

Sexual dimorphisms in weaponry are often the result of intrasexual selection where males compete for resources necessary for reproduction or access to females (Darwin, 1871). Under intrasexual selection, if weapon size can vary seasonally, males would be predicted to invest more in weapon growth during the reproductive season, as we found here in snapping shrimp. However, we also found in females, to the best of our knowledge, the first evidence of the opposite pattern: larger weaponry in the nonreproductive season than in the reproductive season. Increased investment in weaponry outside of the reproductive season is not consistent with intrasexual selection on females, nor can this pattern be explained solely by higher costs to females for producing and carrying the weapon. Larger weaponry in females during the nonreproductive season may result from trade‐offs between investment and reproduction during the reproductive season, and/or advantages associated with female weaponry in nonreproductive contexts.

Females collected in the reproductive season were also larger in body size than those collected in the nonreproductive season, as well as having proportionally smaller claws. Female fecundity increases with female body size in Alpheid snapping shrimp (Corey & Reid, 1991; Costa‐Souza, Rocha, Bezerra, & Almeida, 2014; Knowlton, 1980; Pavanelli, Mossolin, & Mantelatto, 2008,2010), suggesting that differential investment in growth of body size over claw size would be advantageous to females, especially coming into the reproductive season. Costs associated with reproduction may also limit investment in claw size during the reproductive season. Egg production represents a substantial energy investment by females (Gorokhova & Hansson, 2000; Nicol, Mare, & Stolp, 1995) and thus may limit the potential to invest in claw size. Larger relative claw sizes may be additionally disadvantageous for females, due to locomotor or energetic costs of producing and carrying such a substantial appendage. However, in contrast to Hughes et al. (2014), we found no difference in residual claw size between females with and without eggs at capture, and a preliminary analysis of 36 females found no relationship between residual claw size and egg number (Heuring, 2016), suggesting female claw size does not significantly impact reproductive rates or fecundity. Thus, larger body size and proportionally smaller claws during the breeding season are likely to be due to the advantages associated with investing in larger bodies and egg production, rather than costs associated with larger claws per se.

The more intriguing question for females, however, is what are the advantages of larger claws in the nonreproductive season? Indeed, given that body size limits fecundity, one might predict that females would continue to invest differentially in growth of body size during the nonreproductive season, in order to maximize fecundity in the subsequent reproductive season. Two (nonalternative) hypotheses may explain the proportional increase in claw size in the nonreproductive season: differential mortality and differential investment. The decline in body size for females captured in the nonreproductive season suggests higher mortality for large females; if females with proportionally larger claws are at lower risk (independent of body size), differential mortality may contribute to the increase in claw size relative to body size for females captured during the nonreproductive season. Females during the nonreproductive season may also differentially invest in growth of proportionally larger claws.

Either explanation suggests a functional advantage of proportionally larger claws for females that remains unclear. In *A. heterochaelis*, when two females competed for a burrow, winners had larger residual claw sizes than losers, suggesting that proportionally larger claws are advantageous in competitive interactions between females (Hughes, 1996). If females prioritize growth of body size and egg production during the reproductive season, the nonreproductive season may be an opportunity to “catch up” on growth of larger claws, to enhance or maintain competitive ability. Competitive ability may be more critical to females during the nonreproductive season, because the proportion of paired shrimp is significantly lower during this time (W. L. Heuring & M. Hughes, in prep), and single females are more likely than paired females to be evicted by female intruders (Mathews, 2002). If females are more likely to be defending their burrow without male assistance during the nonreproductive season, larger claws may be more advantageous during this time.

In contrast with females, males have larger claws during the reproductive season than the nonreproductive season. Differential investment in larger weaponry during the reproductive season could be advantageous if larger weapons lead to greater success in burrow (and female) defense. Larger weapons would seem an obvious advantage in competitive interactions, and indeed, for many crustaceans (e.g., lobsters (Scrivener, 1971), crayfish (Berrill & Arsenault, 1984; Rutherford, Dunham, & Allison, 1995; Stein, 1976), shore crabs (Sneddon, Huntingford, & Taylor, 1997), and female snapping shrimp, *A. heterochaelis* (Hughes, 1996)), larger claws confer an advantage. Surprisingly, however, we lack direct evidence of a competitive advantage associated with larger claws in male snapping shrimp. Paired and unpaired males do not differ in residual claw size (residuals from the regressions of claw size on body size; Hughes et al., 2014); in the congener *A. heterochaelis*, body size (rather than claw size) predicts the winners of competitive interactions, and winners of competitive interactions do not differ from losers in residual claw size (Hughes, 1996). Nonetheless, males defending a burrow with a female close to molt (and therefore close to being able to mate) are less likely to be evicted by an intruder than males defending an intermolt female (Mathews, 2002), suggesting that male–male competition in a mate‐guarding context may be critical to male reproductive success.

Larger claws during the reproductive season could also be advantageous to males for reasons other than the claw's role as a weapon. First, larger claws could be advantageous as signals, either directed at other males, or as signals used in female mate choice (i.e., advantageous under intersexual selection). Larger claws produce snaps with stronger water jets (Heberholz & Schmitz, 1999; Schein, 1975); the claw is also used as a visual signal in some snapping shrimp species (Hazlett & Winn, 1962; Hughes, 1996), and larger claws for a given body size may provide a deceptive signal of body size (Hughes, 2000). In *A. heterochaelis*, females prefer larger males (Rahman, Dunham, & Govind, 2002; Rahman et al., 2004), although whether claw size influences this choice is not known. Alternatively, if males move between burrows more frequently during the reproductive season in search of mating opportunities and thus are exposed to greater predation risk, larger claws may be favored due to their use in antipredator defense.

Snapping shrimp weapon size is sexually dimorphic year‐round: Male claws were larger than female claws in both reproductive and nonreproductive seasons (Table 1). Nonetheless, the sexually divergent patterns of seasonal variation resulted in more similar claw sizes between males and females in the nonreproductive seasons, and greater sexual dimorphism in the reproductive season. These sex differences in the timing of investment in claw growth suggest different functional advantages to weaponry in males and females. Sexually dimorphic exaggerated weaponry in snapping shrimp, then, is likely not due to weaponry being favored in one sex only, but rather by different mechanisms favoring weaponry in males and females.

## CONFLICT OF INTERESTS

We have no competing interests.

## AUTHORS′ CONTRIBUTIONS

M.H. and W.L.H. together conceived and designed the study, collected field data, and drafted the manuscript. W.L.H. performed the measurements and analyzed the data. Both authors gave final approval for publication.

## Data Availability

Data and analysis code are archived in Dryad (https://doi.org/10.5061/dryad.kc40s9h).

## APPENDIX 1

### DEFINING REPRODUCTIVE VERSUS NONREPRODUCTIVE SEASONS

Reproductively active females were defined as those brooding eggs on their pleopods (i.e., swimmerets) at the time of collection. The percent of reproductively active females per collection date was calculated, and the mean percent reproductively active females across collection dates within each month was used to classify months as reproductive or nonreproductive for subsequent analyses. Only collection dates with more than five females collected were used to calculate the mean for each month to avoid low sample size bias, and months with 5 or fewer shrimp collected were omitted.

### SEASONAL VARIATION IN BODY SIZES OF COLLECTED INDIVIDUALS

For males, body length (*F*_(1,54)_ = 0.953, *p* = 0.333) did not differ between shrimp collected during the reproductive and nonreproductive seasons. For females, however, shrimp collected during the reproductive season were larger (*F*_(1,54)_ = 17.033, *p* < 0.001); the decline in female body size in the nonreproductive season was primarily due to fewer very large individuals.
